# Polyphenols: immunonutrients tipping the balance of immunometabolism in chronic diseases

**DOI:** 10.3389/fimmu.2024.1360065

**Published:** 2024-03-15

**Authors:** Carolina Ferreira, Pedro Vieira, Helena Sá, João Malva, Miguel Castelo-Branco, Flávio Reis, Sofia Viana

**Affiliations:** ^1^ Institute of Pharmacology and Experimental Therapeutics, Faculty of Medicine, University of Coimbra, Coimbra, Portugal; ^2^ Coimbra Institute for Clinical and Biomedical Research (iCBR), Faculty of Medicine, University of Coimbra, Coimbra, Portugal; ^3^ Center for Innovative Biomedicine and Biotechnology (CIBB), University of Coimbra, Coimbra, Portugal; ^4^ Clinical Academic Center of Coimbra (CACC), Faculty of Medicine, University of Coimbra, Coimbra, Portugal; ^5^ Polytechnic Institute of Coimbra, ESTESC-Coimbra Health School, Pharmacy, Coimbra, Portugal; ^6^ Institute of Immunology, Faculty of Medicine (FMUC), University of Coimbra, Coimbra, Portugal; ^7^ Coimbra Institute for Biomedical Imaging and Translational Research (CIBIT)/Institute for Nuclear Sciences Applied to Health (ICNAS), University of Coimbra, Coimbra, Portugal; ^8^ Institute of Physiology, Faculty of Medicine, University of Coimbra, Coimbra, Portugal

**Keywords:** polyphenols, Mediterranean phytochemicals, immunometabolism, immunonutrition, pharmacological immunomodulation, senolytics, immunometabolic diseases, precision nutrition

## Abstract

Mounting evidence progressively appreciates the vital interplay between immunity and metabolism in a wide array of immunometabolic chronic disorders, both autoimmune and non-autoimmune mediated. The immune system regulates the functioning of cellular metabolism within organs like the brain, pancreas and/or adipose tissue by sensing and adapting to fluctuations in the microenvironment’s nutrients, thereby reshaping metabolic pathways that greatly impact a pro- or anti-inflammatory immunophenotype. While it is agreed that the immune system relies on an adequate nutritional status to function properly, we are only just starting to understand how the supply of single or combined nutrients, all of them termed immunonutrients, can steer immune cells towards a less inflamed, tolerogenic immunophenotype. Polyphenols, a class of secondary metabolites abundant in Mediterranean foods, are pharmacologically active natural products with outstanding immunomodulatory actions. Upon binding to a range of receptors highly expressed in immune cells (e.g. AhR, RAR, RLR), they act in immunometabolic pathways through a mitochondria-centered multi-modal approach. *First*, polyphenols activate nutrient sensing via stress-response pathways, essential for immune responses. *Second*, they regulate mammalian target of rapamycin (mTOR)/AMP-activated protein kinase (AMPK) balance in immune cells and are well-tolerated caloric restriction mimetics. *Third*, polyphenols interfere with the assembly of NLR family pyrin domain containing 3 (NLRP3) in endoplasmic reticulum-mitochondria contact sites, inhibiting its activation while improving mitochondrial biogenesis and autophagosome-lysosome fusion. *Finally*, polyphenols impact chromatin remodeling and coordinates both epigenetic and metabolic reprogramming. This work moves beyond the well-documented antioxidant properties of polyphenols, offering new insights into the multifaceted nature of these compounds. It proposes a mechanistical appraisal on the regulatory pathways through which polyphenols modulate the immune response, thereby alleviating chronic low-grade inflammation. Furthermore, it draws parallels between pharmacological interventions and polyphenol-based immunonutrition in their modes of immunomodulation across a wide spectrum of socioeconomically impactful immunometabolic diseases such as Multiple Sclerosis, Diabetes (type 1 and 2) or even Alzheimer’s disease. Lastly, it discusses the existing challenges that thwart the translation of polyphenols-based immunonutritional interventions into long-term clinical studies. Overcoming these limitations will undoubtedly pave the way for improving precision nutrition protocols and provide personalized guidance on tailored polyphenol-based immunonutrition plans.

## 1 Polyphenols: leading-edge immunonutrients

The immune system, a complex interactive network of many different immune cells, mediators, and cellular mechanisms, is highly dynamic in the response to changes in the tissue environment and plays a vital role in the balance between health and disease (1). It generally comprises two lines of defense: the innate (or unspecific) which comprises the physical barriers (e.g. skin, mucosal membranes, commensal microbiota) and several innate immune cells such as neutrophils, macrophages (phagocytes), innate lymphoid cells and nonspecific mediators that rapidly detect antigens, and the adaptive (or specific) immunity that involves B and T cells (2).

Strong evidence links undernutrition to immunosuppression, decreased vaccination efficacy (3, 4) and/or a greater difficulty in recovering from infections, broadly recapitulated during the COVID-19 pandemic (5, 6). On the other hand, overnutrition is closely associated with chronic low-grade inflammation and an increased risk of metabolic diseases (7). Thus, nutritional interventions tagging specific metabolic pathways in immune cells are promising to tackle the increasing prevalence of chronic diseases featuring a dysfunctional immunometabolic status (8), as well as the immunosenescence characterizing the aging process (9).

While it is agreed that immune function relies on an adequate nutritional status to function properly, we are only just starting to understand how the supply of single or combined nutrients, all of them denominated immunonutrients, can redrive the polarization of immune cells towards a tolerogenic or less inflamed immunophenotype (1, 6, 10). Many nutrients fall within the definition of immunonutrients, the most well-known being omega-3 fatty acids, glutamine, arginine, branched-chain amino acids (BCAAs; leucine, isoleucine, valine) and nucleotides (11, 12). Immunonutrition, a branch of precision nutrition, outlines the opportunity to integrate specific nutrients, or foods, in the usual diet (12) and has been drawing the attention of the scientific and medical communities due to its promising health benefits arising from immune system modulation in varied contexts, from individuals undergoing surgical procedures to critically ill patients, subjects with immune-related diseases, the elderly and, in a distinct scope, professional athletes (1, 2). In a multidisciplinary perspective, immunonutrition is defined as the modulation of immune system by nutrients and non-nutritive substances (e.g. antioxidants, prebiotics or probiotics), collectively termed immunonutrients, which are administered in doses above those normally obtained from the diet (1). These molecular compounds display a double function: they act as dietary constituents and, at the same time, may optimize immune responses by improving defense function while maintaining diet and commensal tolerance (1, 12). One may consider immunonutrition as a set of four main mutually dependent concepts: immune system, nutrition, body organ metabolism and the microbiome (1). Besides acting as a physical barrier, the microbiome interacts dynamically with both the innate and adaptive immune system of mucosa-associated lymphoid tissue (MALT) (13). Consequently, it has a chief role in MALT-dependent processes such as oral tolerance induction, cytokine secretion and overall regulation of immune responses. The possibility to reshape microbiota through immunonutrition in the form of functional foods, nutraceuticals and/or dietary supplements, is therefore an exciting approach to switch off oxidative stress and low-grade inflammation present in a plethora of immunometabolic diseases (2, 14).

A wide variety of non-nutritive phytochemicals have shown to benefit immune homeostasis, polyphenols the most-representative ones (12, 15, 16). This group of secondary plant metabolites is a promising class of phytochemicals that hold the potential to simultaneously balance the gut microbiome (14, 17) and the immune system by reprogramming immunometabolic pathways towards the repolarization of immune cells into a tolerogenic, less inflamed phenotype (6). Accordingly, much interest has been created on their potential use as prophylactic or nutritional interventions targeting immunometabolic diseases.

In this work, we aim to shed light on the immunomodulatory effects of polyphenols, leading-edge immunonutrients, on non-communicable chronic diseases that share immunometabolic impairments, both auto-immune and non-autoimmune mediated. It provides a critical appraisal into their capacity to modulate immunometabolic reprogramming, emphasizing polyphenols’ immunomodulatory roles in the maldaptation of organ-specific immune functions as well as their potential use as precise immunonutritional interventions in immunometabolic diseases.

## 2 Polyphenols: dietary sources, structural diversity and bioactivity

### 2.1 Dietary sources

The therapeutic potential of plant-based natural compounds and the phytochemicals composing them has been a significant point of interest in the last years. The most abundant and widely distributed bioactive molecules are polyphenolic compounds (PCs) (18). PCs are significantly abundant in a series of foods including olive oil, herbs, vegetables, fruits, seeds, nuts, whole-grain cereals, and wine that are frequently held accountable for the health benefits of the Mediterranean dietary pattern (19). Each of the referred food groups is enriched in specific PCs classes: phenolic acids predominate in cereals and whole-grains such as wheat, oats, rice, corn, and triticale (20); flavones and hydroxycinnamic acids in dried herbs such as oregano and peppermint (21); catechins, hydroxycinnamic acids, anthocyanins, and proanthocyanidins in red wine (22); flavonoids, phenolic acids and dihydrochalcones in fruits such as apples, mangos and pomegranates for instance (23, 24), and anthocyanins in berries, in which they are responsible for their unique pigmentation and aroma (25). In fact, Mediterranean nutritional patterns are associated with the consumption of colorful meals composed of a high variety of plant-based foods whose sensory and nutritional qualities, namely astringency, color and scent partially derive from the PCs composing them (26, 27).

### 2.2 Chemical structures

Polyphenolic compounds present a phenolic ring as their basic monomer (18). Due to their chemical structure, PCs present strong free radical scavenging capacity which confers them the ability to activate biological antioxidant responses (28, 29). Besides scavenging free radicals, some PCs are also capable of inhibiting the formation and/or activation of their precursors (28, 29). Depending on their chemical structure, origin and biological function, PCs can be divided in different classes, the largest ones being (1) flavonoids and (2) phenolic acids (18). Examples of more narrow classes are (3) tannins, which include pro- and antoanthocyanidins, gallotannins and ellagitannins, (4) coumarins, (5) lignans, (6) quinones, (7) stilbenes, including resveratrol and pterostilbene for instance, and (8) curcuminoids such as curcumin and ginerol analogues (30).

#### 2.2.1 Flavonoids

**Figure 1:**
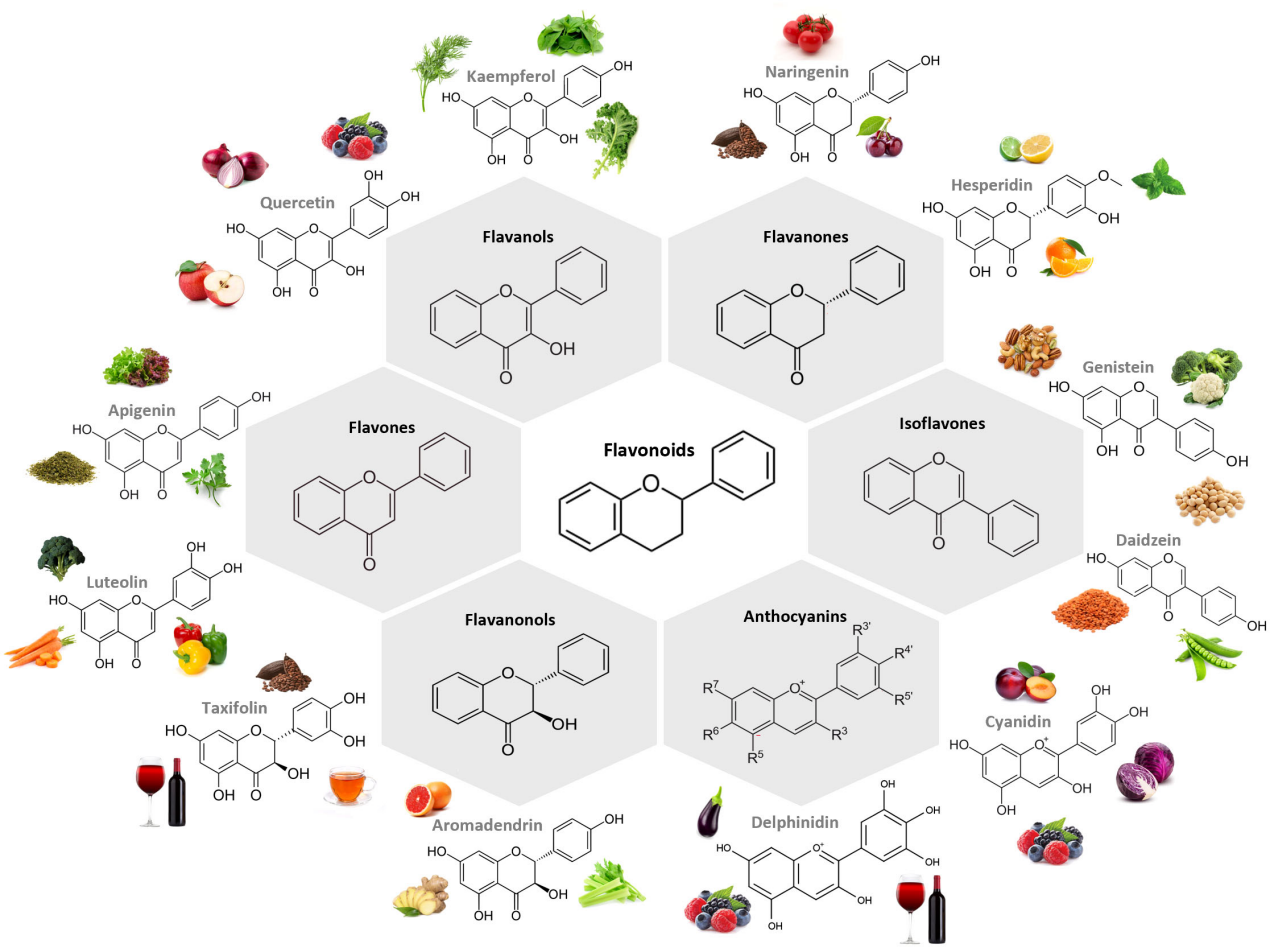
Chemical structure of the main subclasses of flavonoids, examples of compounds belonging to each subclass and examples of foodstuff containing them. The largest subgroup of flavonoids are flavanols, in which the hydroxyl group is positioned in the C3 of the C ring. Flavanones and flavones display a hydroxyl group in the C5 of the A ring with the difference between them residing in the double bond formed between positions 2 and 3, which is saturated in flavanones. Isoflavones differ from flavones on the position of the phenyl group, being structurally similar to estrogens. Flavanonols present the hydroxyl group linked to the C ring in the position 3, and no double bound between this and position 2. Anthocyanin hydroxyl groups of the A and C rings is what dictates their color. All subclasses can be found in fruits and vegetables. Figure created in BioRender.com.

In plants, flavonoids are responsible for the coloring and aroma of flowers and fruits (24) and the majority are found as glycosides (18). The general structural backbone of flavonoids is C6–C3–C6, the carbon of the C ring on which the B ring is attached to being the determinator of the subgroup the compound belongs to (18, 24). When the link between the B and the C rings is in the position 3, they are isoflavones, and when this link happens in position 4 we stand before neoflavonoids (24). Those in which the B ring is attached to the C one in the position 2 are further classified into different subgroups depending on the structure of the C ring, them being flavanones, flavanonols, flavones, flavonols, flavanols and anthocyanins (24). **Figure 1** presents the chemical structure of the most common compounds belonging to flavonoid subclasses.

#### 2.2.2 Phenolic acids

**Figure 2:**
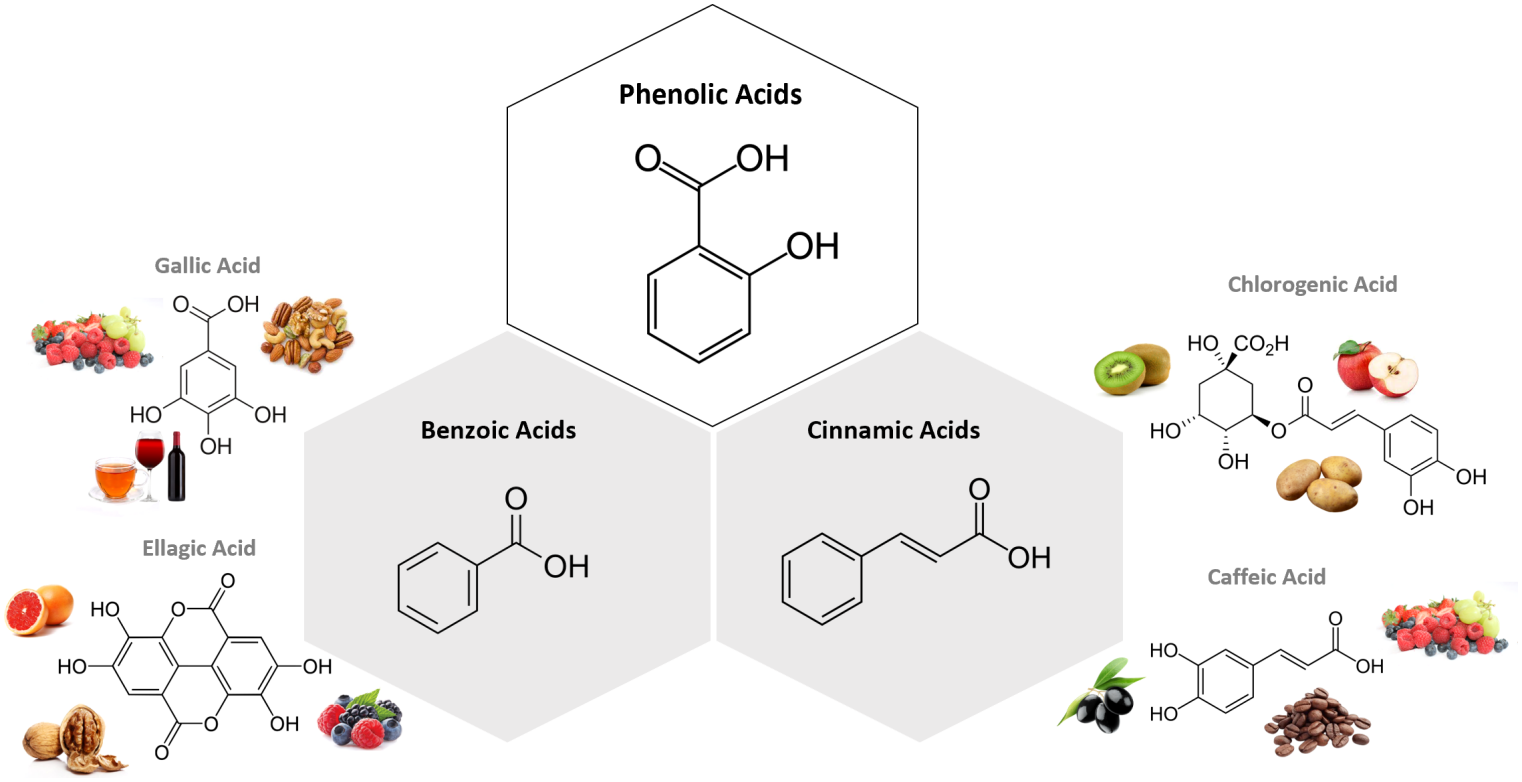
Chemical structure of the subclasses of phenolic acids, examples of compounds belonging to each subclass and examples of foodstuff containing them. The most abundant hydroxycinnamic acid found in food is chlorogenic acid (CGA), which is an ester formed between caffeic and quinic acids. On another hand, the most common hydroxybenzoic acids are gallic, vanillic, ellagic, syringic, p-hydroxybenzoic, and protocatechuic acids. Figure created in BioRender.com.

Phenolic acids are PCs that possess one carboxylic acid group, and can be divided into two major subtypes: benzoic acids, which present a skeletal structure C6-C1, and cinnamic acids, whose structure is C6-C3 (31). They are present in innumerous plant-based foods, such as fruits, vegetables, seeds, legumes, cereal and coffee, being mainly in a bound form, such as amides, esters and glycosides (31). The most abundant hydroxycinnamic acid found in food is chlorogenic acid (CGA), which is an ester formed between caffeic and quinic acids (31). On another hand, the most common hydroxybenzoic acids are gallic, vanillic, ellagic, syringic, p-hydroxybenzoic, and protocatechuic acids (31). These compounds might act as neuroprotective agents through radical-scavenging activity, being useful in the context of chronic diseases associated with oxidative stress (31). **Figure 2** presents the chemical structure of the most common compounds belonging to phenolic acids subclasses.

### 2.3 Bioavailability and bioactivity

The overall bioavailability of PCs is determined mainly by their chemical structure, their absorption, distribution, metabolism, excretion (ADME), the form of administration, and food matrix (32). Pharmacokinetic studies show that PCs classes vary in terms of bioavailability and can be placed as follows: phenolic acids > isoflavones > flavonols > catechins > flavanones, proanthocyanidins > anthocyanins (32–34).

The polyphenolic content of several plants and fruits is greatly affected by exogenous factors (e.g. climatic conditions, culture types, the degree of ripeness), storage, cooking methods and processing mechanisms (33). Besides, food related factors such as the presence of specific macro and micronutrients can as well modify PCs bioavailability and bioactivity (35). Interestingly, it has been recently suggested that the association between PCs and fiber delays their absorption through the gastrointestinal (GI) tract, potentially optimizing their assimilation (36).

To exert their bioactivity, PCs must be delivered to the GI and absorbed, reach circulation and, posteriorly the target tissues, being subjected to a significant degree of transformation along their journey through the GI tract. As consequence, a single PC is able to generate several different metabolites displaying different activities and properties relatively to the original compound. In general, PCs display low oral bioavailability (5-10%) (37), due to factors such as decreased solubility, the interaction with the food matrix, difficulties in membrane-crossing, as well as their extensive hepatic and intestinal metabolism and rapid clearance (32). Still, they display a plethora of scientifically proved and extensively documented dose-dependent beneficial effects (38). Accumulating evidence on the health-promoting effects of many PCs make them a topic of interest for scientists, nutritionists, and consumers in general. Their advantageous features, including marked antioxidant, anti-inflammatory, antimicrobial and anti-adipogenic properties grant them great potential to be incorporated in functional foods, nutraceuticals and/or dietary supplements (**Table 1**). Notably, PCs are closely intertwined to the therapeutic potentiation of the immune system, adding an extra layer of complexity to their pleiotropic actions. Polyphenols immunomodulatory and anti-inflammatory activities correlate to the number, positions and types of substitutions as well as the degree of polymerization based on the chromane ring (53). Moreover, the high degree of hydroxylation in the B-ring of cathecins and anthocyanidins favor metabolic reprogramming and polyphenols’ bioactivity (54). In the upcoming section, the impact of polyphenols in immunometabolic reprogramming of both innate and adaptive immune responses will be discussed.

**Table 1:** Bioactivity of polyphenolic compounds.

Bioactivity		Polyphenolic compound		Evidence		Reference	
**Antioxidant**		Catechins		Catechins displayed the most favorable results regarding a series of antioxidant activity evaluation assays		(39)	
		(-)-Epigallocatechin-3-gallate from Green Tea		Stimulate nuclear factor erythroid 2–related factor 2 (Nrf2) translocation to the nucleus		(40)	
		Kaempferol, Gallic Acid, Resveratrol		Potentiate the activity of enzymes belonging to the endogenous biological antioxidant system, such as catalase, superoxide dismutase, glutathione peroxidase and glutathione-S-transferase		(41–43)	
**Anti-inflammatory**		Hesperidin		Reduce interleukin (IL)-6, tumor necrosis factor alpha (TNF-α) and nitric oxide levels both *in vitro* and *in vivo*		(44)	
		Dehydroxylated Phenolic Acids: 3,4-dihydroxyphenylpropionic acid, 3-hydroxyphenylacetic acid, 4-hydroxyhippuric acid		Attenuate lipopolysaccharide (LPS)-induced secretion of TNF-α, IL-6 and IL-1β in human peripheral blood mononuclear cells (PBMCs)		(45)	
**Antimicrobial**		Epigallocatechin Gallate, Tea Polyphenols, A-type Proanthocyanidins		Disturbing the cell wall of specific bacteria, their inner cytoplasmatic membrane, or reducing their motility and biofilm-forming ability		(46–48)	
**Anti-adipogenic**		Vanillic Acid, Catechins, Resveratrol		Adipose tissue “browning” Suppress the expression of genes and transcription factors related to adipogenesis		(49–52)	

## 3 Polyphenols and immunometabolic reprogramming: a multi-organelle approach

Immunometabolic reprogramming heavily relies on inter-organelle communication and mitochondria, key organelles for cellular metabolism, act as masters regulators of multi-organelle connections and immune cell-fate determination (55, 56). During the immune response, cells shift from metabolic quiescence to an active phase, and the preferential utilization of specific metabolic pathways can dictate immune cells’ differentiation towards a pro- or anti-inflammatory immunophenotype depending on their specialization for mounting protective immunity or tolerance to self or external antigens (57).

The interaction among nutrient signaling networks, adenosine triphosphate (ATP) availability, and immunological cues is crucial to meet the energy demands and functional modifications in immune cell metabolism. AMPK and its downstream target, mTOR, serve as central hubs to nutrient availability by sensing intracellular energy levels (AMP/ADP: ATP ratio). In energy-depleted states, activated AMPK typically inhibits mTOR signaling and promotes mitochondrial biogenesis via the peroxisome proliferator-activated receptor-gamma (PPARγ) co-activator-1 alpha (PGC1α) signaling axis (58). Consequently, cellular metabolism skews towards increased oxidative phosphorylation (OXPHOS) activity and enhanced expression of genes encoding key mitochondrial enzymes. Conversely, in states of overnutrition, mTOR upregulates protein and lipid synthesis to promote immune cell growth and proliferation (59).

Quiescent immune cells, such as naïve T cells, memory T cells (Tmem), Treg or tolerogenic DCs, alongside M2 macrophages, predominantly favor mitochondria-driven catabolic metabolism characterized by OXPHOS and fatty acid oxidation (FAO) to sustain ATP supply for long-term survival (60, 61). Autophagy, a conserved lysosomal degradation pathway that supports immune cell differentiation, is enforced by AMPK activation, thereby restraining glycolysis and maintaining cellular quiescence (7, 62). Contrariwise, activated immune cells, such as M1 macrophages and effector T cells (e.g. Th1, Th17), exit the quiescence state by metabolizing nutrients to ensure an adequate supply of macromolecules for the energy demands associated with cellular growth (55). They shift the balance towards mTOR activation and aerobic glycolysis as a rapid source of ATP, akin to the Warburg effect, to meet the high nutritional and energetic requirements of short-term clonal expansion and effector function (4, 10, 60). For example, mTORC1 sustains aerobic glycolysis and upregulates hypoxia-inducible factor 1 alpha (HIF-1α) expression to support Th17 cell differentiation, counteracting Treg expansion (58). Similarly, a significant transition from OXPHOS to aerobic glycolysis occurs in bone marrow-derived DCs upon Toll-like receptor (TRL) activation, resulting in inducible nitric oxide synthase (NOS)-dependent generation of nitric oxide (NO) and blockade of mitochondrial electron transport (61). Metabolic reprograming of activated immune cells also involves glutaminolysis. Glutamine is converted into glutamate and ketoglutarate, two well-known tricarboxylic acid cycle (TCA) intermediates that support the oxidative metabolism of immune cells, particularly macrophages. A high ketoglutarate/succinate ratio promotes alternative (M2) activation and FAO engagement, while a low ratio strengthens the proinflammatory phenotype observed in classically activated (M1) macrophages (63).

Metabolic rewiring entails significant modifications in mitochondrial biogenesis and dynamics, as well as redox signaling pathways, all of which are crucial for immune function. For instance, the immunometabolism of T cells heavily relies on the continuous dynamic reshaping of mitochondria through fusion and fission events to maintain mitochondrial quality. Memory T cells undergo increased mitochondrial fusion to support OXPHOS and fatty acid oxidation (FAO) metabolism. In contrast, activated effector T cells demonstrate heightened rates of mitochondrial fission and reduced cristae, an adaptation to facilitate aerobic glycolysis (64). Membrane-bound organelles such as mitochondria, endoplasmic reticulum (ER) and lysosomes must establish inter-organelle connections through specialized cytosolic microdomains to facilitate the intersection of metabolic signaling and the utilization of products from one pathway efficiently as intermediates for another (65). ER-mitochondria junction signaling provides a regulatory platform for various overarching immune cellular functions. The mitochondria-ER network brings together signaling components to potentiate mitochondria fission and Warburg metabolism, key events for the rapid recall response of newly activated memory CD8+ T cells (66, 67). Similarly, the activation of NLRP3 spatially correlates to mitochondria-derived reactive oxygen species (mtROS) and excessive mitochondrial fission in ER-mitochondria contact sites of macrophages undergoing glycolytic reprogramming (64, 68, 69). In summary, the dynamic behavior of mitochondria and inter-organelle communication, particularly with the ER network and endolysosomal system, is crucial for enabling immune cells to seamlessly adjust to fluctuations in nutrient availability. This aptitude is vital for effectively meeting the functional demands during immune cell remodeling.

**Figure 3:**
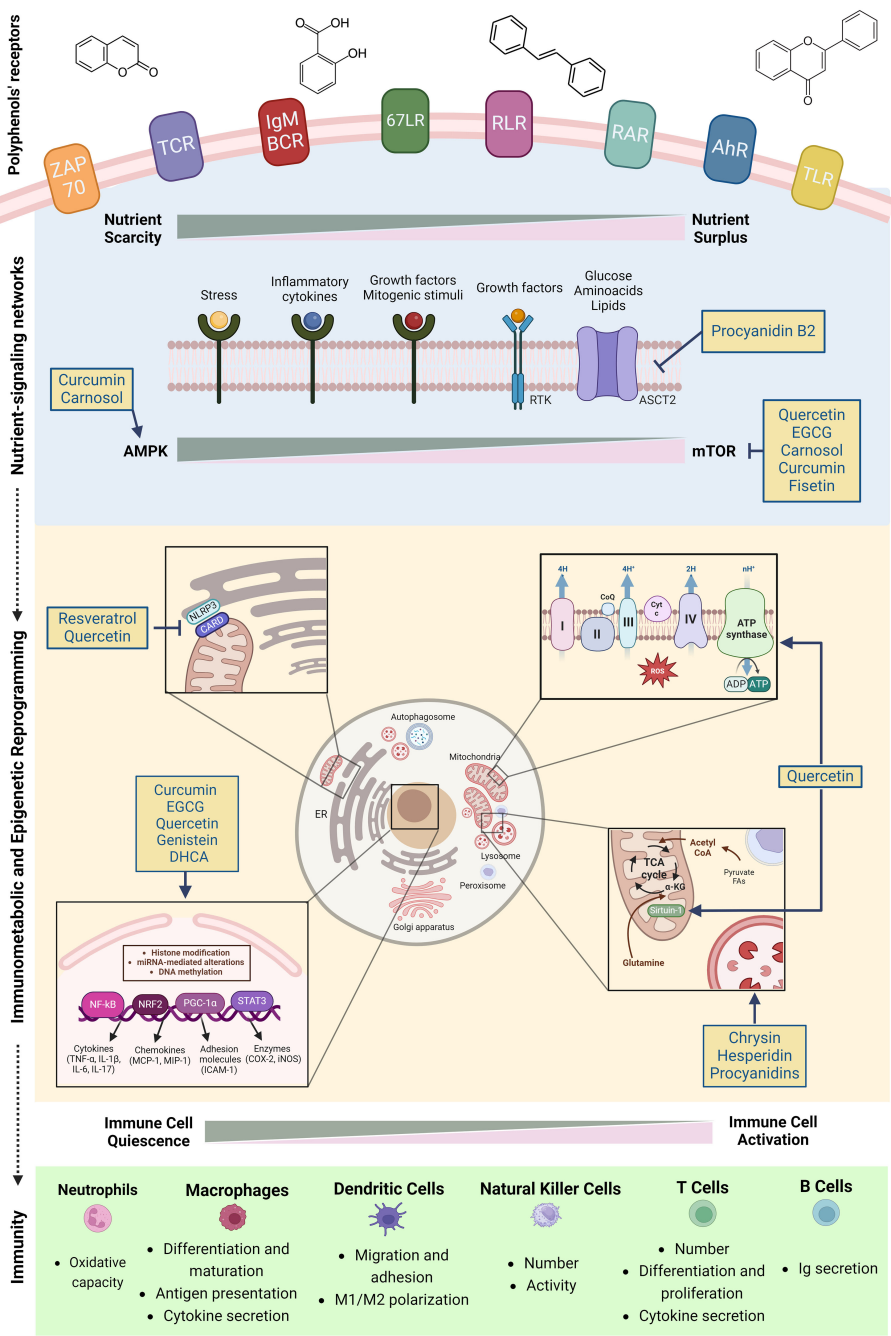
Polyphenols and immunomodulation: a mitochondria-centered multi-modal approach. Polyphenols impact immunometabolic reprogramming through four regulatory axes: first, they activate nutrient sensing via stress-response pathways and growth factors, essential for immune responses. Second, polyphenols regulate mTOR/AMPK balance and inflammatory responses in immune cells and serve as well-tolerated caloric restriction mimetics. Third, they interfere with the assembly of NLRP3 in endoplasmic reticulum-mitochondria contact sites, inhibiting its activation while improving mitochondrial biogenesis and autophagosome-lysosome fusion. Finally, polyphenols impact chromatin remodeling through modulation of histone deacetylase/acetyltransferase, thereby coordinating both epigenetic and metabolic reprogramming. Figure created in BioRender.com.

Evidence regarding the immunomodulatory effects of PCs have been significantly emerging in the last decades (8, 70, 71). A main reason relies on the fact that different immune cell populations express various kinds of polyphenols’ receptors (72). Examples of immune cellular receptors targeted by PCs include the retinoic acid-inducible gene like receptors (RLRs), aryl hydrocarbon receptor (AhR), 67 kDa laminin receptor (67LR), zeta chain-associated 70 kDa protein (ZAP-70), T cell receptor (TCR) αβ, secretory IgM- (sIgM-) B-cell receptor, Toll-like receptor (TLR) 4 (73–79) and Retinoic Acid Receptors (RARs) (80, 81). Upon binding, PCs are able to modulate immune cells metabolism and activity through a multi-modal approach encompassing nutrient-sensing mechanisms, AMPK/mTOR signaling balance, regulation of inter-organellar communication and modulation of metabolism-epigenetic axis (**Figure 3**).

### 3.1 Nutrient-sensing mechanisms

Nutrients not only act as building blocks but also activate nutrient sensing via stress-response pathways and growth factors, essential for immune responses (82). Under amino acid starvation conditions`, immune cells activate the amino acid response (AAR)`, a cytoprotective signaling pathway that transiently reduce protein synthesis while enhancing stress-induced gene expression (83). AAR pathway is a potent regulator of inflammatory T cell differentiation. Accordingly, glutamine uptake and glutaminolysis largely cooperate in Th1/Th17 inflammatory T cell response (84). Glutamine, the most abundant amino acid in human plasma, is an important substrate of various ATP generating pathways (e.g. glycolysis, OXPHOS) (85). It is transported across the plasma membrane in mammalian cells by different transporters such as the alanine serine cysteine transporter 2 (ASCT2). Gallate-type procyanidin PCB2 3,3 (PCB2DG) polyphenol, a dimer of epicatechin, interacts directly with ASCT2 glutamine transporter and antagonizes glutamine influx, mTOR/HIF-1 pathway, Th1/Th17 cell production and inflammatory response through interferon gamma (IFNγ) and interleukin-17 (IL-17) production.

Sirtuin-1 (SIRT1) serves as another crucial energy sensor. It is activated by NAD+ in nutrient-deficient states and modulates mitochondrial biogenesis by deacetylating and activating transcription factors such as PCG-1α, signal transducer and activator of transcription 3 (STAT3) or the nuclear factor E2-related factor (NRF)-2. In CD4+ T cells, SIRT1 impedes the process of differentiation of T lymphocytes into Th17 cells through STAT3 deacetylation (86). Therefore, SIRT1 agonists have emerged as promising pharmacological approaches to broaden the array of current therapeutic options focused on reducing the Th17 profile. Among these possibilities, the deacetylase activator resveratrol stands out as particularly promising. In CD4+ T cells, this polyphenol has been observed to encourage Th2 and Treg polarization, immunomodulatory effects that are linked to the diverse beneficial impacts of resveratrol in various pathologies characterized by imbalanced lymphocyte subtypes ratios (87).

### 3.2 AMPK/mTOR signaling balance

mTOR and AMPK stand out as two additional master regulators of cellular metabolism, enabling adaptation to challenges of nutrient scarcity or excess, ultimately promoting cell survival. They are intricately linked to cell-specific adjustments in response to metabolic stress, and disruptions in these signaling pathways are closely associated with various pathological conditions (88). When nutrients are abundant, organisms prioritize fuel utilization to support cellular growth, with mTOR signaling playing a central role in this process. Conversely, upon nutrient depletion, organisms suppress anabolic pathways and promote autophagy via AMPK signaling to adopt a state geared towards preserving the structural and functional integrity of existing cells. Importantly, SIRT1/PGC1α can exert negative regulation on the phosphoinositide 3-kinase (PI3K)-alpha serine/threonine-protein kinase/(Akt/mTOR) pathway, likely through their influence on the cellular maintenance of autophagy (89). Senolytic drugs can simultaneously upregulate nutrient deprivation signaling (AMPK) and suppress pathways associated with nutrient surplus (mTOR), consequently boosting autophagic flux (90). Accordingly, caloric restriction mimetics are the most extensively studied metabolic interventions and have long been associated to lifespan extension and immunosenescence improvement (91, 92).

PCs are well-tolerated caloric restriction mimetics due to their ability to activate AMPK, a cellular energy sensor, thus improving mitochondrial turnover (93, 94). Quercetin and fisetin, two well-established senolytic drugs, belong to the flavonoid class of polyphenols and are key modulators of immune cell function. In lipopolysaccharide (LPS)-treated macrophages, fisetin inhibited PI3K/AKT/mTOR signaling and inflammatory cytokines secretion (95). In addition, the acetyltransferase inhibitor epigallocatechin-3-gallate (EGCG) was found to downregulate mTOR-HIF1α signaling, a metabolic checkpoint of Th17/Treg differentiation, leading to the downregulation of glycolysis-associated molecules and inhibition of Th17 differentiation (70). Likewise, carnosol and curcumin effectively inhibit mTOR activation in response to LPS stimulation in human DCs via AMPK-dependent induction of heme oxygenase-1 (HO-1), an important antioxidant enzyme that assist the maintenance of DCs in a tolerogenic state (71).

### 3.3 Mitochondria and ER-lysosomes inter-organellar communication

The NLRP3 inflammasome, a critical junction between innate and adaptive immunity, relies on ER-mitochondria contact sites to facilitate the association of mitochondria-driven ligands, including dysfunctional mitochondria themselves, as upstream signals for NLRP3 activation. Additionally, self-derived or foreign-derived particulates can be endocytosed by lysosomes, leading to membrane damage and further release of cathepsin B, another common upstream signal for NLRP3 activation (96). In LPS-treated microglia cells, quercetin enhances the mitophagic clearance of damaged mitochondria, countering mtROS accumulation and NLRP3 inflammasome activation during the assembly stage (97). Similarly, resveratrol inhibits the acetylated α-tubulin-mediated spatial arrangement of mitochondria and their ER contacts in macrophages. Consequently, it interferes with the assembly of NLRP3 and its adaptor protein, apoptosis-associated speck-like protein containing a caspase recruitment domain (ASC), thereby inhibiting NLRP3 inflammasome activation triggered by mitochondrial damage (98).

Interestingly, it has been proposed that polyphenols can be directly endocytosed into lysosomes, regulating key signaling pathways of phagocytic cells such as macrophages and DCs (99). Accordingly, chrysin (a flavone) and hesperidin (a flavonoid) enhanced lysosomal phosphatase activity in a concentration-dependent manner in LPS-stimulated macrophages (100). Similarly, the senolytic drug fisetin facilitates the autophagosome-lysosome fusion and degradation processes in LPS-treated macrophages by regulating a set of genes primarily involved in autophagosome assembly/maturation (95). Comparable effects were observed in LPS-treated DCs, where cocoa procyanidins strongly upregulated gene pathways associated with lysosomal metabolic function and nutrient metabolism, suggesting a significant impact on DC metabolic activity (99).

### 3.4 Modulation of metabolism-epigenetic axis

Recent research has revealed that alterations in metabolic status can coordinate the function of immune cells by influencing epigenetic changes. This regulatory axis between metabolism and epigenetic enables the microenvironment to mold immune cells, and disruption of this process can contribute to the development of various diseases (101). For example, in LPS-stimulated THP-1 promonocyte cells, TLR4 stimulation triggers glucose-dependent ATP production alongside gene-specific chromatin remodeling. Sirt1 deacetylation activates PGC1α transcriptional activity and orchestrates sequential metabolic reprograming, sensing processes dependent on NAD+, thereby reducing HIF1α-dependent glycolysis and enhancing PGC1α-dependent FAO (102). Notably, quercetin upregulates Sirt1/PGC1α signaling and improves mitochondrial function and morphology (e.g. mtROS, mitochondrial membrane potential, ATP production) in LPS-induced inflammatory macrophages (103).

Chromatin remodeling involves structural changes such as DNA methylation, histone methylation, and acetylation, which greatly impact transcriptional changes of different genes. Several polyphenols have been identified as histone deacetylase (HDAC) inhibitors (EGCG, curcumin, genistein, quercetin), histone acetyltranferase (HAT) activators (genistein) or HAT inhibitors (EGCG, curcumin) (104). For instance, gallic and ellagic acids, along with fisetin, were found to decrease HAT activity in THP-1 cells, resulting in the deacetylation of the p65 subunit of NF-κB and attenuation of pro-inflammatory cytokine release (105, 106). Moreover, EGCG enhances HDAC activity in Treg cells, leading to suppressed nuclear factor kappa-light-chain-enhancer of activated B cells (NF-kB) signaling and elevated synthesis of the anti-inflammatory IL-10 (107). Finally, treatment with dihydrocaffeic acid (DHCA) led to a decrease in DNA methylation levels in peripheral leukocytes from mice exposed to stressful conditions as well as human and mice peripheral leucocytes exposed to lipopolysaccharide (LPS) *in vitro* (108).

## 4 Polyphenols and immunomodulation

### 4.1 Polyphenolic modulation of innate immunity

In a simplified manner, one might consider that innate immunity includes two distinct components: the cellular system and the non-cellular system (70). As its name suggests, the cellular system is composed of a set of different cell populations, such as granulocytes, monocytes, macrophages, natural killer and dendritic cells (70). On another hand, the non-cellular one includes diverse kinds of mechanisms that range from mucous barriers to signaling pathways (70). Both components act in a synergistic manner in order to prevent pathogens’ access to the organism or promote their destruction in case the referred barriers have already been broken (70).

#### 4.1.1 Effects of polyphenols on dendritic cells

Due to their antigen-presenting activity, dendritic cells (DCs) are indispensable for initiating and regulating innate immune responses (70). PCs have been showing to influence several aspects related to DCs, including differentiation and maturation, as well as their antigen presentation and cytokine secretion functions. For instance, resveratrol was shown to regulate the differentiation of healthy human monocytes from the blood into DCs (109). Analogously, both EGCG and quercetin exerted immunosuppressive effects in bone marrow (BM)-derived DCs, impairing their maturation and their expression of major histocompatibility complex (MHC) (110, 111). An *in vitro* study has shown that quercetin’s inhibition of DC maturation results from downregulated steroid receptor coactivator (Src)/PI3K-Akt-NF-kB-inflammatory pathways (112). Furthermore, EGCG exposure induced apoptosis of blood monocyte-derived DCs from healthy individuals and modulated developing DCs’ phenotype by downregulating MHC II molecules and the surface markers CD11c, CD80 and CD83, which are needed for the process of antigen presentation (113). Interestingly, polyphenols of different natures have been shown to possess immunosuppressive properties towards murine BM-derived DCs stimulated with LPS, including curcumin (114), apigenin (115), daidzein (116), baicalin (117), fisetin (118) and silybin (119). These PCs significantly inhibited the expression of surface markers associated with DC maturation such as CD40, MHC II molecules, as well as costimulatory receptors namely CD80 and CD86, in a dose-dependent manner. As a consequence, they impacted the induction of Th1-mediated immune responses. Additionally, the referred study employing curcumin has also reported a decreased production of IL-1β by DCs, once more repressing their immunostimulant activity (114). Many of these effects seem to derive from the polyphenols’ ability to modulate DC metabolism, namely through suppressing mitogen-activated protein kinases (MAPKs) p38, c-Jun-N-terminal kinase (JNK), extracellular regulated kinase (ERK) 1 and 2, and NF-κB activation (111, 114, 115, 119, 120). Analogously, carnosol and curcumin were found to affect AMPK activation and downstream inhibition of the mTOR pathway in lipopolysaccharide-prime DCs (121). The reduced glycolytic flux promoted by the two polyphenols also impacted mitochondria, inhibiting the LPS-induced increase of spare respiratory capacity.

#### 4.1.2 Effects of polyphenols on monocytes and macrophages

Similarly to DCs, macrophages play an important role in antigen presentation mechanisms, as well as tissue inflammation and repair processes (70). Remarkably, the shift between M1 and M2 phenotypes has shown to be influenced by PCs. For instance, *in vitro* culturing of THP-1 macrophages with a cocoa extract resulted in suppressed M1-mediated inflammation and promoted polarization to M2 (122). A similar effect has been observed with resveratrol regarding tumor-associated macrophages (123). Moreover, quercetin, kaempferol, daidzein, genistein (124) and apigenin (125) have exhibited the ability to reduce pro-inflammatory cytokines’ secretion by these cells. Quercetin has shown to prevent the secretion of IL-6, IL-1β and tumor necrosis factor alpha (TNF-α) by macrophages by suppressing LPS-induced MAPK and ERK activation (126). Plum polyphenols have also been linked to decreased pro-inflammatory cytokines, ROS and malondialdehyde production by RAW 264.7 macrophages treated with monosodium urate through different signaling pathways involving HIF-1, ErbB and Forkhead box transcription factor O (FoxO) (127). A similar effect has been reported for hesperidin which besides decreasing *ex vivo* IL-12 secretion in LPS-stimulated mouse macrophages also suppressed their migration and adhesion properties *in vitro* (128). An interesting study aiming to evaluate the impact of the flavonoids quercetin, naringenin and naringin on the metabolism of cultured human macrophages has highlighted that the flavonoid-mediated immunomodulation derived from glycolytic downregulation, as well as anti-inflammatory reprogramming of the TCA cycle and antioxidant protection (mainly quercetin), membrane modification (naringenin) and osmoregulation (naringin) (129).

PCs are also able to modulate macrophagic ROS production and iNOS activity, as has been reported for curcumin (130, 131), resveratrol (132, 133), EGCG (134, 135), and genistein (136), to name a few.

Polyphenols further seem to improve macrophages’ phagocytic capacity. EGCG and curcumin, for instance, have been shown to trigger murine peritoneal macrophages and RAW 264.7 macrophages’ phagocytosis *in vitro* (137, 138). The synergistic effect of these two polyphenols together with resveratrol has been demonstrated against glioblastoma and human papillomavirus (HPV)-infected cells, leading to the repolarization of tumor-associated macrophages and tumor suppression (139).

Interestingly, PCs seem to not only influence macrophages but also their precursors -monocytes – as evidenced by an increase in nitric oxide (NO) production by blood monocytes observed in healthy individuals consuming red wine (72). Moreover, blueberry supplementation has been shown to decrease monocyte expression of monocyte-to-macrophage differentiation-associated (MMD) and C-C motif chemokine receptor 2 (CCR2), reducing inflammation in metabolic syndrome patients (140). EGCG prevented monocyte adhesion to cultured endothelial cells from pig pulmonary aortas by reducing the expression of vascular cell adhesion molecule-1 (VCAM-1) and monocyte chemotactic protein-1 (MCP-1) (141).

#### 4.1.3 Effects of polyphenols on neutrophils

As has been observed for DCs and macrophages, studies highlighting the immunomodulatory effects of PCs on this cell population have been arising, particularly regarding their ability to inhibit *in vitro* neutrophils’ oxidative capacity, which correlates with exacerbated neutrophilic inflammation (142, 143). Accordingly, a study performed by Drábiková et al. reported that a series of polyphenols including curcumin, pinosylvin, resveratrol, pterostilbene, piceatannol and N-feruloylserotonin significantly reduced ROS production by human neutrophils *in vitro* (144). Furthermore, human blood cultured neutrophils’ exposed to treatment with grape polyphenols exhibit improved chemokinetic accuracy and motility in association with enhanced CD16 shedding and CD66b expression (145). On another hand, a study evaluating the impact of phenolic acids in a mouse model of colitis exalted the ability of ferulic acid to alleviate the disease by suppressing the formation of neutrophil extracellular traps (146).

#### 4.1.4 Effects of polyphenols on natural killer cells

Natural Killer (NK) cells are recognized by their robust cytotoxicity and lytic activity, as well as effector functions (147). Contrarily to what has been described above regarding the effects of PCs on macrophages, DCs and neutrophils, which are essentially immunosuppressive, their impact on NK cells appears to have a stimulatory nature, increasing their number and activity. As an example, green tea polyphenols and quercetin are able to promote murine NK-mediated cytotoxicity (148) and lytic activity (149), respectively. Similarly, low-dose resveratrol supplementation has promoted NK cell killing capacity in different experimental contexts (150–152), potentially by activating JNK and ERK (152, 153). Nevertheless, this seems to be dose-dependent since high doses of resveratrol exerted the opposite effect. Similarly, a study performed by Oo et al. reported that luteolin, apigenin and quercetin at doses of 12.5 µg/ml and 25µg/ml significantly increased the NK-cell-mediated cytotoxic activity against lung cancer cells (154). Contrastingly, genistein blocks NK cells’ activity at low doses (155) but enhances their cytotoxicity at high concentrations (156). These results highlight the dose-dependent behavior displayed by the vast majority of polyphenolic compounds.

In humans, clinical studies showed that blueberry supplementation increases NK cell count in the blood of healthy subjects (157, 158).

### 4.2 Polyphenolic modulation of adaptive immunity

Alternatively to the innate immune system, the adaptive branch of the immune system involves a unique type of cells - lymphocytes (70). Two primary lymphocyte populations prevail (1): T lymphocytes, which are responsible for cytokines’ secretion, cytotoxic destruction of unviable cells and activation of other immune cells, and (2) B lymphocytes, known by their antibody-producing capacity (70).

#### 4.2.1 Effects of polyphenols on T and B cells

The immunomodulatory potential of PCs goes beyond innate immunity, considerably impacting lymphocyte numbers and functionality. For instance, incorporating EGCG in the diet for one week has proven to elevate T regulatory (Treg) cells’ number in mice’s spleen, mesenteric and pancreatic lymph nodes (159). Furthermore, these cells were able to repress cytotoxic T cell action and proliferation as well as interferon gamma (IFNγ) production (159). A study evaluating EGCG’s impact on naïve CD4^+^ T cell differentiation showed that the green tea polyphenol inhibited Th1, Th9, and Th17 differentiation by downregulating the respective transcription factors T-bet, PU.1, and RORγt, while also preventing IL-6-induced suppression of Treg development. These effects were considered to result from downregulation of Signal transducer and activator of transcription p-STAT1 and p-STAT4 for Th1, and p-STAT3 for Th17 cells, as well as inhibition of IL-6-induced STAT3 phosphorylation, respectively. Analogously, naringenin displayed the potential to induce Treg cells through AhR-mediated pathways (72) and baicalin has shown to inhibit Th17 cell differentiation both *in vitro* and *in vivo* via reducing RAR-related orphan receptor gamma t (RORγt) expression and up-regulating Forkhead box p3 (Foxp3) expression (160). Interestingly, EGCG has also shown to induce Treg cells by repressing DNA methylation, inducing Foxp3 and IL-12 expression both *in vitro* and *in vivo* (159). These outcomes exalt a novel epigenetic mechanism underlying the polyphenol’s immunomodulatory activity associated with DNA methyltransferases inhibition. Moreover, Ning et al. provided new evidence for the effectiveness of the green tea flavonoid in vitiligo treatment via Janus kinase 2 (JAK2) kinase activity inhibition, reducing the protein levels of CD11a, CXCR3, and CCR2 receptors in human T lymphocytes, suppressing their adhesion to melanocytes induced by IFN-γ (161). Importantly, EGCG’s immunomodulatory properties are not limited to CD4^+^ T cells. In fact, there are several reports on the flavonoid’s competence on increasing CD8^+^ T cell number and activity in tumorigenic contexts (162, 163). Genistein has exhibited a similar effect, while also enhancing CD8^+^ T cell IFNγ expression both *ex vivo* and *in vivo*, leading to immune stimulation (156).

A study performed by Ramiro-Puig et al. evaluating the effects of a cocoa-enriched diet in the spleen lymphocyte function of young rats reported that a 10% cocoa intake increased lymphocyte proliferation rate, but down-regulated Th2-associated cytokine levels and decreased immunoglobulin (Ig) secretion (164). Additionally, spleen B cell proportion was raised, and Th cell percentage declined (164).

Similarly, auraptene, a citrus fruit-derived coumarin, was able to suppress the activation of murine inguinal lymph node-derived Th1 cells (165). Finally, genistein has also shown to increase the number of both helper and cytotoxic T cells as well as B lymphocytes in rat spleen (166). Likewise, curcumin administration to Min/+ mice increases mucosal CD4^+^ T and B cell numbers by modulating CD28, CTLA-4, STAT and NF-kB expression, preventing the formation of intestinal tumors (167). In addition, through inhibiting STAT4 phosphorylation curcumin has also shown to suppress human CD4^+^ T cells differentiation into the Th1 phenotype (168). Curiously, curcumin’s impact appears to depend on the stimulous to which lymphocytes have been exposed, since other studies exalt its immunossupressive activity. For instance, Sharma et al. reported that both resveratrol and curcumin suppressed the activity of concavilin A-stimulated T and B cells by inhibiting their proliferation, antibody production and lymphokine secretion (169). In fact, curcumin’s ability to suppress B cell proliferation has also been demonstrated in human Epstein-Barr infected cells (170). Curiously, polyphenol-driven apoptosis of leukemic B cells was shown to correlate with caspase 3 activation, reduced mitochondrial transmembrane potential as well as downregulation of antiapoptotic protein beclin 2 and iNOS expression (171).

## 5 Polyphenol-based immunonutrition in immunometabolic diseases

Over the past two decades, the pivotal interplay between immunity and metabolism in chronic diseases has become increasingly evident (172). The burgeoning field of Immunometabolism has progressively illuminated how the immune system orchestrates the functionality of key homeostatic systems within tissues, such as the brain, pancreas, liver and adipose tissue. This modulation occurs through the sensing and adaptation to microenvironmental nutrient fluctuations, driving flexibly reprogramming of metabolic pathways in immune cells that greatly impact their polarization towards a pro- or anti-inflammatory phenotype (172, 173). Accordingly, mounting body of evidence progressively appreciates the mobilization of the innate and adaptive immune systems not only in autoimmune diseases featured by the loss of self-tolerance but also in supposedly non-immune pathologies encompassing neurodegeneration and metabolic disorders (174). Consequently, there’s a rising interest in immunonutritional approaches aimed at optimizing immune cells functions to enhance effective defense responses while preserving tolerance.

**Figure 4:**
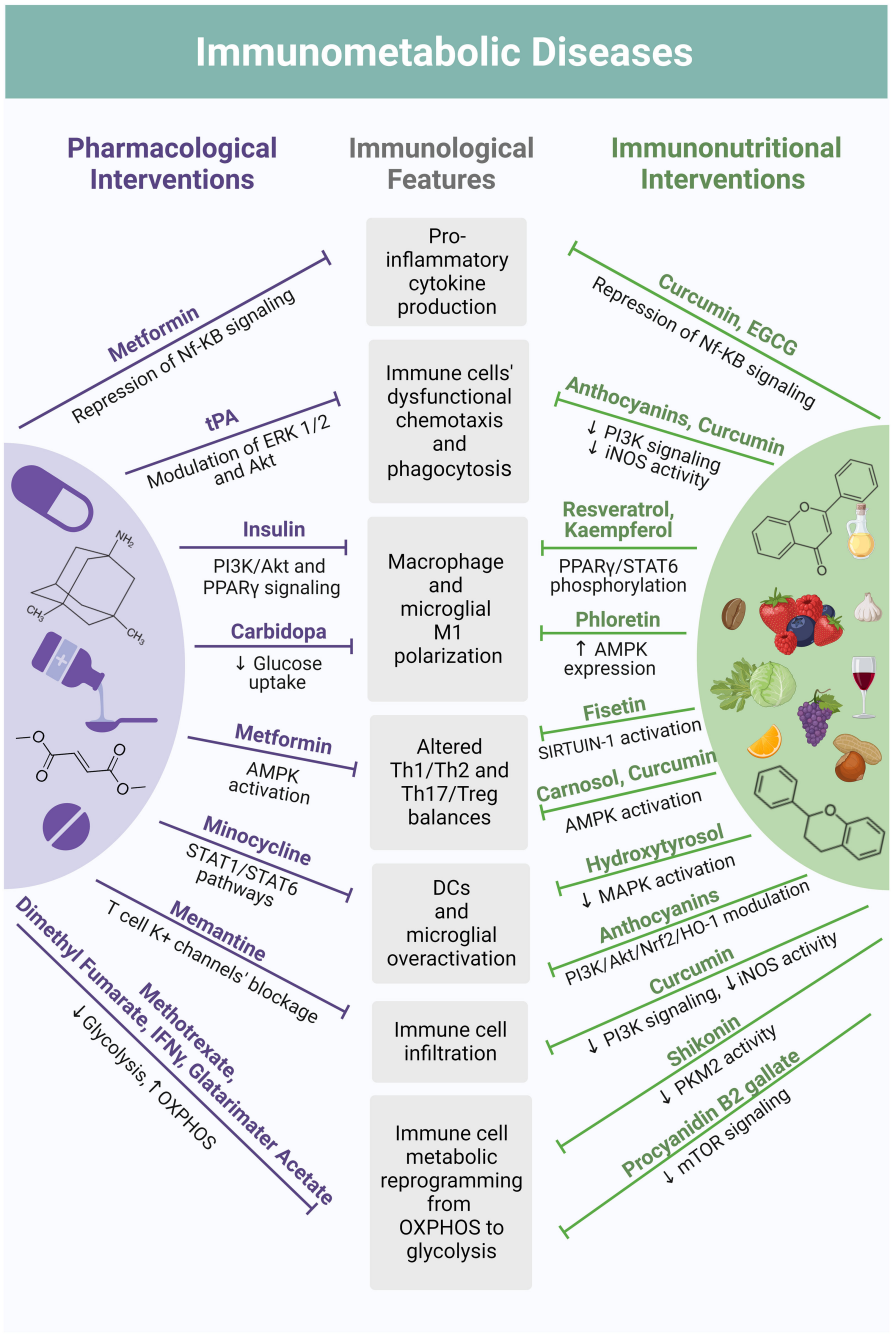
Mechanistical parallelism between pharmacological interventions and polyphenol-based immunonutrition in their modes of immunomodulation. Current therapeutic algorithms of immunometabolic disorders include drugs with pleiotropic immunomodulatory actions shared by a plethora of polyphenol-based immunonutritional approaches. For instance, the anti-diabetic metformin suppresses the production of pro-inflammatory cytokines by immune cells through Nf-KB signaling modulation, the same effect being reported for curcumin and the flavonoid EGCG. The anti-diabetic has also displayed the ability to counteract unbalances in T cell subpopulations by activating AMPK, an effect once again shared by the polyphenol’s curcumin and carnosol. Tissue plasminogen activator (tPA), a serine protease used in stroke therapy, was found to improve chemotaxis and phagocytic ability of immune cells through metabolic pathways’ modulation, including ERK 1/2 and Akt signaling. An equivalent effect has been reported for curcumin and anthocyanins, due to suppression of PI3K signaling and iNOS enzymatic activity. Through these same mechanisms, curcumin also attenuates immune cell infiltration, exerting an effect similar to the NMDA receptor antagonist memantine, an anti-dementia drug widely used in Alzheimer’s disease, which blocks T cells’ potassium channels. Minocycline, a tetracycline antibiotic currently being studied as a therapeutic strategy for stroke, prevents DCs and microglia cells from excessive activation by modulating the JAK/STAT signaling pathway. Likewise, anthocyanins are suggested to modulate the PI3K/Akt/Nrf2/HO-1 axis in DCs and microglia cells, suppressing their overactivation. This is also achieved with the polyphenol hydroxytyrosol, which reduces MAPK activation. A similar mechanism has been observed for polyphenols belonging to different classes, such as resveratrol and kaempferol, which also regulate PPARγ, inhibiting macrophage and microglia polarization towards a pro-inflammatory (M1) phenotype. These polarization-shifting properties are likewise reported for the flavonoid phloretin and are considered to be mediated by an increased AMPK expression. These effects are analogous to the ones of insulin, which is directed to T1DM patients and interferes with PI3K/Akt and PPARγ signaling, and to the decarboxylase inhibitor carbidopa, which promotes M2 macrophage polarization in the context of Parkinson’s Disease by suppressing glucose uptake. Under environmental stimuli, immune cell activation occurs accompanied by metabolic reprogramming. In most cases, this primarily consists of a transition from mitochondrial OXPHOS to aerobic glycolysis. Drugs purposed for Multiple Sclerosis treatment, such as dimethyl fumarate, IFNγ and glatarimater acetate, among others, are known to modulate this shift, suppressing glycolysis and promoting OXPHOS in T cells. The same is observed for methotrexate – an antimetabolite used to treat IBD. Analogously, by interfering with mTOR signaling and PKM2 activity, flavonoids such as shikonin and procyanidin B2 gallate, respectively, also modulate immune cell metabolic reprogramming in the context of immunometabolic dysfunctions. Figure created in BioRender.com.

The following sections delve into the characteristics of immunological disturbances within the spectrum of both auto-immune and non-autoimmune metabolic disorders (175). Additionally, it sheds light on the immunomodulatory roles of polyphenols and draws a mechanistical parallel between their effects and the pleiotropic immunomodulatory actions of drugs currently integrated into corresponding therapeutic algorithms (**Figure 4**).

### 5.1 Autoimmune immunometabolic diseases

#### 5.1.1 Type 1 diabetes mellitus

Type 1 diabetes mellitus (T1DM) is currently accepted as being a T cell-mediated disease (176). Nevertheless, other adaptive immune cells as well as elements from innate immunity are believed to be involved in T1DM physiopathology.

Namely, T1DM patients display an impaired complement system function (177) and monocytes from these patients display decreased chemotaxis and phagocytic activity (178). Furthermore, infiltration of macrophages (179),neutrophils, and NK cells (180) in the pancreatic Langerhans islands has been detected in NOD mice and human patients. Moreover, hyperglycemia has shown to impair macrophages’ autophagic mechanisms (181, 182). Interestingly, insulin has shown to reestablish the normal phenotype in diabetogenic macrophages through Akt and ERK signaling (183), as well as to repress TLRs and CD14 transcription (184). Furthermore, Yu et al. reported insulin’s ability to promote phenotype transition of macrophages from M1 to M2 through PI3K/Akt pathways, and PPAR-γ signaling during diabetic wound healing (185).

Nonetheless, cellular infiltrates found on the pancreas of diabetic subjects are also composed of adaptive immune cells, such as CD4^+^ and CD8^+^ T as well as B lymphocytes (176). Remarkably, diabetogenic CD4^+^/CD8^+^ T lymphocytes are more dependent on aerobic glycolysis and rely less on OXPHOS (186). Accordingly, glycolysis inhibition induced terminal CD4^+^ T cell exhaustion in an animal model of T1DM, delaying disease onset (187). Treg cells are also found to be dysfunctional in the pancreatic lymph nodes of T1DM patients (188) and an increase in IL-17-producing T cells has also been detected (188, 189). Diabetogenic T cells are further characterized by mitochondrial membrane hyperpolarization and dysfunction, resulting in increased ROS levels and diminished ATP production (190).

PCs supplementation has been emerging as a potential therapeutic strategy for alleviating the immune dysfunction characterizing T1DM, in part by improving mitochondrial function. In fact, several kinds of polyphenols have shown to improve mitochondrial function, namely through the activation of the key mitochondrial biogenesis’ PGC-1α, including ursolic acid (191), resveratrol (192), quercetin (193, 194) and olive hydroxytyrosol (195).

Nevertheless, PCs’ effects are not limited to mitochondrial function. For instance, a pomegranate peel extract was able to inhibit immune cell infiltration into pancreatic islets (196). Similarly, oral administration of capsaicin to several mice strains showed to attenuate the proliferation and activation of autoreactive T cells in pancreatic lymph nodes, protecting them from disease development (197). The authors considered these effects to be mediated by capsaicin-mediated enhancement of a discreet population of CD11b^+^/F4/80^+^ macrophages in the pancreatic lymph nodes, which express the anti-inflammatory factors interleukin IL -10 and programmed death-ligand 1 (PD-L1). Moreover, procyanidin B2 gallate has been revealed as a suppressor of TNF-α production by activated CD4^+^ T cells by inhibiting their glycolytic function via mTOR- HIF-1α interaction (198). Lastly, a study evaluating the impact of black seeds and garlic intake in diabetic rats demonstrated a significant increase in the blood levels of monocytes and granulocytes, while lymphocyte proliferation was suppressed (199). A similar output was verified when administering fenugreek oil to a rat model of T1DM, which blunted the diabetes-induced increase of pancreatic lymphocyte counts (200).

#### 5.1.2 Inflammatory bowel disease

Inflammatory Bowel Disease (IBD) presents defects in peripheral and intestinal immune function (201). A deep analysis of the peripheral immune system of IBD patients has found decreased numbers of NK cells and B lymphocytes opposing to increased counts of neutrophils and memory CD8^+^ T cells in the blood (202). Besides displaying elevated phagocytosis and cytokine production (201), IBD-associated macrophages also go through the Warburg effect by HIF-1α stabilization and subsequent increased expression of glycolytic enzymes, a process that is modulated by pyruvate kinase 2 (PKM2) (203). The disease further entails gut DCs overactivation, resulting in increased levels of IL-6in the serum and intestine of IBD patients. Decreased numbers of Treg cells in mice peripheral blood and patients’ intestinal mucosa, alongside the expansion of Th17 cells and increased production of IL-17 and IL-23 in the intestinal mucosa and lamina propria (LP) have also been detected (201). Interestingly, the expression of pro-inflammatory cytokines by Th17 cells was found to be epigenetically controlled by the glucose transporter GLUT3, which is upregulated in models of IBD (204). Collectively, evidence points to a relevant role of glycolysis in the immunologic dysfunction characterizing IBD. Accordingly, therapy with methotrexate, which is used for different autoimmune conditions including IBD, appears to suppress glycolytic mechanisms in varied immune cell populations (205), counteracting the metabolic reprogramming associated with disease pathophysiology.

Furthermore, intestinal barrier function is also impaired in the context of the disease, presenting less mucus secretion by goblet cells, reduced antimicrobial peptides (AMPs)’ production by Paneth cells and several mutations in genes coding tight junction proteins, resulting in their dysfunction and consequent loss of barrier integrity (201). Defects in mucosa mitochondrial function are also a feature of IBD, including reduced complex I activity, membrane potential, biogenesis, OXPHOS, TCA cycle and fatty acid metabolism alongside increased mitochondrial fragmentation due to fission (206, 207).

Furthermore, the disease is characterized by increased susceptibility to dysfunctional autophagy of macrophages, DCs, Paneth cells and GCs (208). Particularly, Autophagy Related 16 Like 1 (ATG16L1) gene deficiency in macrophages increases the risk of Chron’s Disease development (209) and suppresses DCs’ ability to induce Treg cells in contexts of intestinal inflammation (210).

Remarkably, a part of PCs’ beneficial effects regarding IBD is related to their impact on autophagy. As an example, the flavonoid galangin has shown to alleviate DSS-induced colitis’ symptoms in mice by increasing the expression of autophagy-related proteins and promoting colonic autophagosome formation (211). On another hand, resveratrol displayed autophagy-promoting properties in cultured macrophages through sirtuin modulation (212, 213), highlighting its potential to counteract the macrophagic autophagy dysfunction underlying IBD.

PCs further display relevant potential to maintain intestinal homeostasis by protecting the intestinal barrier. Several studies evaluating the impact of polyphenolic supplementation in experimental models of the disease have revealed improved gut barrier function, consequently limiting inflammatory cell infiltration. This has proved to be true for grape seed PCs which increase colonic goblet cell density and mucin 2 mRNA expression (214); anthocyanins by enhancing tight junction molecules (zonulin-1, claudin-1, occludin) and Muc 1/2 expressions (215), just to mention a few. Interestingly, resveratrol and resveratrol-related PCs (e.g. pterostilbene) have further demonstrated to alleviate intestinal inflammation in mice with colitis by regulating the Th17/Treg balance and control the levels of plasmatic and intestinal mucosal cytokines such as transforming growth factor beta (TGF-β), IL-6, IL-10 and IL-17 (216, 217), restoring the percentage of CD4^+^ T cells in mesenteric lymph nodes (MLNs) and decrease their number in the intestinal LP, as well as reducing the percentage of macrophages in both regions (218). Similarly, curcumin appears to promote colonic Treg cell expansion while decreasing the counts of inflammatory DCs; inhibiting pro-inflammatory cytokines’ secretion, T cell infiltration and NF-kB activation (219), as well as to suppress macrophage activation and regulate M1/M2 polarization (220). Chlorogenic acid has also shown to mitigate DSS-induced colitis in mice by inhibiting M1 macrophage polarization through suppressing PKM2-dependent glycolysis and Nod-like receptor protein 3 activation (146).

Moreover, a study performed by Wu et al. reported that the lignan arctigenin inhibits Th17 and Th1 differentiation *in vitro* by repressing STAT3 and STAT4 phosphorylation respectively through mTORC1 downregulation, ameliorating DSS-induced colitis in mice (221).

On another hand, shikonin – a polyphenol widely used in Chinese traditional medicine – has shown to suppress glucose consumption and lactate production as well as inhibit the nuclear translocation and enzymatic activity of PKM2, which is responsible for stimulating the Warburg effect in macrophages, in a DSS-induced colitis mouse model (222).

Furthermore, PCs are known for inducing short-chain fatty acids (SCFAs) production by the gut microbiota, namely butyrate (223, 224), which displays several gut health-promoting properties: it promotes colonic mucus production (225); potentiates the extrathymic conversion of CD4-positive T lymphocytes into Treg cells; is able to reduce mTOR activation and glycolysis in intestinal macrophages, while simultaneously promoting their metabolic reprogramming to OXPHOS and lipid metabolism (203) as well as downregulating their expression of pro-inflammatory cytokines (226). Remarkably, some of the aforementioned effects equally emerge from methotrexate therapy for CD, which elevates OXPHOS in T cells by activating AMPK and blocking mTORC1 (227).

In humans, consumption of mango by IBD patients significantly improved Simple Clinical Colitis Activity Index (SCCAI) score and decreased the plasma levels of pro-inflammatory cytokines related to neutrophil-induced inflammation (228).

#### 5.1.3 Multiple sclerosis

Similarly to T1DM and IBD, Multiple Sclerosis (MS)’ pathophysiology is characterized by a series of immunological alterations, the most well-known pathophysiological components of the disease (229). Although the primary events leading to the autoimmune attack characterizing MS are not yet established, a possible explanation is based on molecular mimicry consisting of the activation of autoreactive T lymphocytes through cross-reactivity by viral and/or bacterial antigens structurally similar to central nervous system (CNS) proteins, such as myelin basic protein (MBP), myelin oligodendrocyte glycoprotein (MOG) and proteolipid protein (PLP) (229). These cells migrate to the CNS fueling neuroinflammatory events that promote BBB opening allow a second wave of immune cells to access the CNS, namely CD8^+^ T cells, B lymphocytes and macrophages (230). Macrophages within the perivascular cuff of post-capillary venules of animals with EAE display altered metabolism featured by increased expression of the glycolytic enzyme lactate dehydrogenase (LDHA) as well as monocarboxylate transporter-4 (MCT-4), specialized in secreting lactate from glycolytic cells, potentially inducing macrophage infiltration in the CNS (231). Analogously to what is described for T1DM, activated CD4^+^ T cells from MS patients display an up-regulation of aerobic glycolysis and down-regulation of OXPHOS (232), as well as altered mitochondrial structure, mitochondrial DNA (mtDNA) levels and membrane potential (233). Furthermore, abnormal expression of autophagy-related markers and genes has been found in T cells from MS patients and EAE animals (234). It is worth noting that the role of autophagy in MS pathophysiology is controversial since there is evidence of both protective and deleterious effects of autophagy induction in immune cells in the context of the disease (234), but there seems to be a consensus regarding the fact that autophagy contributes to MS pathology in macrophages, DCs, T and B cells while having a protective role in neurons and glial cells (235).

On another hand, there is a decreased count of circulating Treg cells in MS patients (236), which also seem to play an important role in EAE development (237). In fact, the immunometabolism of T cells is extremely relevant in the pathophysiology of MS, as evidenced by the fact that several of its therapeutic strategies modulate T cell metabolic features: Dimethyl Fumarate suppresses glycolysis; IFNγ decreases ATP levels, mitochondrial membrane potential and modulates OXPHOS; Teriflunomide limits T cell activation by blocking mitochondrial respiratory chain’s complex III; and Glatiramer Acetate promotes OXPHOS and represses glycolysis in CD4^+^ T cells (238). Likewise, a study evaluating the effects of cinnamic acid in EAE reported that the polyphenol acted as an MCT-4 inhibitor, attenuating immune cell infiltration into the CNS, suppressing glycolysis and lactate production by macrophages and ultimately reducing disease severity (231). The EAE-associated inflammatory phenotype of macrophages has also been reduced by the dihydrochalcone phloretin, which activated Nrf2 by stimulating AMPK-dependent autophagy (239). Additionally, a large number of PCs have shown to attenuate EAE clinical severity or inhibit its development by reducing immune cell infiltration, referring to EGCG (240), curcumin (241) and hesperidin (242). The latter two also seem to impact the Th17/Treg balance in EAE animals, promoting Treg cell expansion and Th17 suppression in the spleen (241), lymph nodes (240, 242) and the CNS, accompanied by repressed pro-inflammatory cytokine secretion (240, 241). Analogously, EGCG as well as naringenin for instance are known to impact the Th1-mediated immune response associated with EAE (240, 243). The described outcomes are potentially attributed to the impact of PCs on the expression of transcription factors associated with each of the referred T cell subsets: Foxp3 for Tregs (242, 243), RORγt for Th17 cells (241–243), and T-bet for Th1 ones (243). Inhibition of Th17 cells’ differentiation by curcumin further entails down-regulating IL-6 and IL-21 as well as STAT3 phosphorylation (241). Considering that MS is a T cell-mediated disorder, inhibition of CD4^+^ T cells’ activation might comprise a promising therapeutical strategy. Interestingly, curcumin has shown to induce human T cell death through increased expression of ER stress-related transcriptional factors (244). Analogously, resveratrol inhibits CD4^+^ T cells’ activation and cytokine production by promoting SIRT1 expression and activity both *in vitro* and *in vivo* (245).The stilbene has further been highlighted as able to counteract the decline in brain mitochondrial function characterizing the cuprizone-induced demyelination model by enhancing cytochrome oxidase activity and elevating ATP levels (245).

### 5.2 Non-autoimmune immunometabolic diseases

#### 5.2.1 Obesity and type 2 diabetes mellitus

Findings arising from pre-clinical and clinical research have been elucidating the mechanisms of immunological dysfunction associated with obesity and type 2 diabetes mellitus (T2DM). Regarding innate immunity, metabolic dysfunctions are characterized by an altered neutrophil functionality, increased M1 macrophage and inflammatory DCs numbers, and abnormal NK phenotypes (246). In obese individuals, neutrophils display augmented chemotaxis and non-directed migration, as well as increased basal levels of superoxide, while neutrophils from diabetic subjects lose a variety of their functions, including migration capacity, phagocytosis and ROS production (246). By increasing leptin levels, obesity alters adipose tissue macrophages’ (ATMs) metabolism through Janus kinase 3 (JAK3) and STAT3, and PI3K-Akt-mTOR pathways, increasing glycolytic enzymes’ activity and glucose uptake as well as inducing mitochondrial dysfunctions (247). PI3K-Akt-mTOR activation in brain macrophages of diabetic rats has further been implicated in autophagy impairment (248), which originates protein aggregates and fosters damaged mitochondria due to defective mitophagy (249). Dysfunctional mitochondria accumulation leads to increased ROS production and consequent NLRP3 dependent-inflammation by macrophages in both T2DM (250) and obesity (251). Mitochondrial dynamics are likewise affected in the context of both disorders, as evidenced by induced activation of the fission regulator dynamin-related protein 1 (Drp1) by a high-fat diet (252) as well as increased mitochondrial fission and decreased fusion in leukocytes from T2DM patients (253).

NK cells are also dysfunctional in contexts of obesity and T2DM, displaying increased proliferation rates and IFNγ secretion, and impaired degranulation, respectively (246). A dysfunctional mTOR function has also been observed in NK cells from obese patients (254). Furthermore, DCs activation and maturation is promoted in cases of diabetes, and obesity-associated DCs present an inflammatory phenotype triggering Th17 cells’ activation (246).

Adaptive immunity is likewise affected by metabolic impairments, resulting in increased numbers of γδ T, Th17 and Th22 cells and a reduction in Tregs (246). B cells display altered functionality, promoting an abnormal antibody response (246). In obesity, CD4^+^ T cells also reveal a distinct metabolic profile characterized by the activation of glycolysis and OXPHOS (255). Furthermore, mitochondria from T2DM patients’ CD8^+^ T cells display higher oxidative capacity together with elevated ROS levels and fatty acid uptake as well as decreased FAO and AMPK activity (256). In fact, metformin, a widely used oral antidiabetic, has shown to facilitate T cells’ shift from a glucose-dependent anabolic state to a catabolic one through mTOR signaling blockage and by restoring mitochondrial FAO (257). Furthermore, it shuts down glycolysis and promotes OXPHOS by activating pathways involving carnitine palmitoyltransferase (CPT)-1 alpha and PGC-1α (258).

The impact of PCs in immune system dysfunctions associated with obesity and T2DM have been consistently highlighted in both *in vitro* and *in vivo* experiments. Analogously to metformin, a variety of polyphenol formulations as well as isolated compounds are described as PGC-1α inducers in the context of T2DM and obesity, such as ginger polyphenols 6-gingerol and 6-chrysophanol (259), epicatechin-enriched cocoa (260), sudachitin (261), and EGCG (262). The latter has also shown to inhibit T2DM-associated mitochondrial deficiency and dysfunction in diabetic Goto-Kakizaki rats by suppressing enhanced autophagy in muscle cells (263, 264). Furthermore, resveratrol administration to older adult diabetics showed to improve mitochondrial biogenesis and function through SIRT1 upregulation, alleviating the oxidative damage and promoting insulin sensitivity (265).

On another hand, PCs that include capsaicin, curcumin, and anthocyanins for instance, have shown to attenuate macrophage migration (266–268), in part by suppressing MCP-1 expression (268, 269). Curcumin has also displayed relevant suppressive effects on NF-kB signaling in immune cells, leading to a reduction in iNOS expression by macrophages and DCs (270), as well as neutrophils (271). Neutrophils chemotaxis is also apparently impacted by curcumin’s ability to suppress PI3K activity and Akt phosphorylation (271). On another hand, evidence suggests that PCs present in the small fruit lingonberry promote macrophage polarization to an anti-inflammatory (M2) phenotype by upregulating PPARγ and STAT6 phosphorylation in experimentally induced obesity (272). Quercetin has also shown to abolish NLRP3 inflammasome activation in macrophages by upregulating Akt signaling, reducing insulin resistance in mice with particulate matter-induced metabolic disorder (273). A similar effect has been reported for red raspberry polyphenols (274).

Regarding adaptive immunity, studies employing cafeteria diet-induced obesity as well as the alloxan-induced model of diabetes in rats have reported that PCs intake lowered the production of pro-inflammatory mediators including ILs, TNFα, IFNγ and TGF-β by MLN and splenic lymphocytes (275, 276). Contrasting to what is observed for macrophages, PCs seem to promote Treg cell recruitment, namely through elevation of Foxp3 gene expression (277). A study employing EGCG in the context of murine diet-induced obesity has reported an increased Treg/Th17 cell balance by decreasing the ratio of STAT3/STAT5 expression (278).

#### 5.2.2 Neurological diseases

Evidence from genome-wide association studies highlight the association between immune cells-mediated inflammation and increased risk of neurodegeneration (279). Most neurodegenerative diseases involve deposition of misfolded proteins, leading to aggregate formation and consequent neuronal loss (279). The initial phases of the referred disorders are characterized by the activation of the immune system and neuroinflammation, partially mediated by a CNS resident macrophage cell population – microglia – that are activated in virtually all neurodegenerative conditions (279). Despite the pivotal role of microglia cells, infiltrations composed of astrocytes, monocytes and/or lymphocytes are also frequent in these contexts (279). Immune cell dysfunctions in the mitochondrial respiratory chain are likewise preponderant features of neurodegenerative diseases, evidence suggesting that leucocytes, neutrophils, monocytes/macrophages, and T cells display increased levels of ROS and NO accompanied by elevated mitochondrial membrane potential and decreased complex activity (238).

Considering the above, previously mentioned reports of PCs ability to limit immune cell activation and cellular infiltration point to these natural substances as promising therapeutic agents in the context of neurological disturbances.

##### 5.2.2.1 Alzheimer’s Disease

Alzheimer’s Disease (AD) is the most common neurodegenerative disease worldwide (279, 280). Its main features include β-amyloid protein (Aβ) deposition and tau protein hyperphosphorylation, originating senile plaques and neurofibrillary tangles (NTFs), respectively (279, 280). The referred aggregates promote microglial activation, which surprisingly appears to play a dual role in disease pathophysiology (280). Initially, activated microglia seem to have a beneficial effect by phagocytizing excessive Aβ, but as the disease progresses, they may lose this ability and acquire a dysfunctional senescent phenotype or become neurotoxic by remaining chronically activated (280). The inflammatory mediators produced by these cells stimulate an analogous response on astrocytes, resulting in neuronal death (280). Furthermore, brain parenchyma infiltration by neutrophils and NK cells also seems to contribute to the neuroinflammatory changes reported in AD (279, 280). On another hand, the role of adaptive immune system in the disease is controversial, since there are studies supporting a neuroprotective role for the adaptive immune cells in AD animal models (281) while others exalt its requirement for disease progression (282). Noticeably, numerous lines of evidence on the impact of metabolic perturbations in microglia mediated-neuroinflammation in AD have been arising. Actually, the age-related decline in glucose metabolism in the brain is associated with cognitive dysfunctions in AD patients (283). Aβ deposition seems to induce mTOR phosphorylation and HIF-1α expression by microglia, originating inflammatory cascades (283). Microglial cells adopt a neuroimmunomodulator phenotype, exhibiting ineffective glycolysis, and TCA cycle accompanied by impaired chemotaxis and phagocytic ability (283). Furthermore, AD features a great degree of mitochondrial dysfunction with concomitant cardiolipin exposure, leading to increased microglial phagocytosis and synthesis of inflammatory mediators, fostering neuroinflammation (284). Moreover, damaged mitochondria release mtDNA which can induce the NLRP3 inflammasome and the NF-kB pathway, exacerbating inflammation (284). AD-associated microglia further display a suppressed autophagic flux due to a reduced expression of key regulatory proteins such as Beclin-1 (285).

Interestingly, Chen et al. has reported that microglial cells mediate T cell infiltration in experimental models of AD as well as human brains, driving neuroinflammation (286). Accordingly, memantine – a drug approved for the treatment of advanced AD – acts on T cell metabolism by blocking potassium channels, normalizing these cells’ response (287).

Likewise, PCs exhibit vast potential as therapeutic agents in neurological pathologies by acting on different strands of their etiology, among which are immune dysfunctions. Interestingly, oleuropein aglycone – a polyphenol abundantly present in extra virgin olive oil – is reported to induce autophagy in AD mouse models by modulating AMPK signaling (288, 289) as well as sirtuin activity and histone acetylation (288). Likewise, curcumin has shown to downregulate PI3K, phosphorylated Akt and mTOR protein levels, inducing autophagy in brain tissue of APP/PS1 double transgenic AD mice (290).

Resveratrol, which is currently under investigation in several clinical trials for AD, has demonstrated to possess remarkable immunomodulatory properties in immune cell populations highly relevant in the context of the disease. It inhibited microglia activation, proliferation and cytokine production (IL-6 and TNF-α) (291) and promoted its polarization towards an anti-inflammatory phenotype in animal models (292), suppressing neuroinflammation. Furthermore, a retrospective study with AD patients demonstrated that treatment with resveratrol induced adaptive immune responses, increasing IL-4, fibroblast growth factor (FGF) 2 and macrophage-derived chemokine (MDC) secretion by macrophages (293). Still regarding microglia cells, anthocyanins were also able to mitigate oxidative stress and neurodegeneration in a mouse model of AD by modulating the PI3K/Akt/Nuclear factor erythroid 2-related factor 2 (Nrf2)/heme oxygenase 1 (HO-1) axis (294). Additionally, supplementation of cultured microglia exposed to Aβ with a polyphenol abundantly found in extra virgin olive oil named hydroxytyrosol attenuated mitogen-activated protein kinases (MAPKs) activation as well as ROS generation (295). The flavonoid baicalein has also shown to inhibit microglia-induced neuroinflammation in a mouse model of AD by suppressing NLRP3 activation and the TLR4/NF-kB pathway (296). Similarly, a study performed by Kim et al. described gallic acid’s profile as a histone acetyltransferase inhibitor, highlighting its ability to inhibit NF-kB acetylation and reducing cytokine production by cultured microglia (297).

##### 5.2.2.2 Parkinson’s Disease

On the frequency ranking for neurodegenerative diseases, Parkinson’s Disease (PD) follows AD at second place and is characterized by the accumulation of α-synuclein in neurons, glial cells, and nerve fibers (279). The histopathological hallmarks of PD include loss of dopaminergic neurons in the *substancia nigra pars compacta* (SNpc), presence of activated microglia, astrogliosis and lymphocytic infiltration (279).

Similarly to what happens in AD, the accumulated protein aggregates promote microglia activation, which proceed to release excessive amounts of neurotoxic factors generating a self-amplifying cycle that contributes to progressive neuronal degeneration (279). PD-associated microglia also display impaired mitochondrial function associated with mutations in genes involved in mitophagy and oxidative stress such as Pink1 and Parkin, resulting in inflammasome activation that fosters dopaminergic neurodegeneration (298, 299). Interestingly, research in PD experimental models has shown that NLRP3 inflammasome activation is exacerbated in microglia cells deficient in autophagy related protein 5 (ATG5) (300), highlighting autophagy’s relevance in suppressing inflammation. Nevertheless, the role of other immune cells such as astrocytes and NK cells in this inflammatory cascade remains to be enlightened (279, 280). On another hand, the expansion of dysfunctional monocytes appears to be an essential element in PD pathogenesis and might be related to the secretion of inflammatory mediators by microglia cells as well as pro-inflammatory monocytes’ recruitment to the brain, fomenting neuroinflammation (279, 280). Furthermore, evidence suggest that M1 macrophages’ activation is linked to disease susceptibility and progression (301). Accordingly, the decarboxylase inhibitor carbidopa that is used for PD management has been shown to favor macrophage differentiation to an M2 phenotype (302).

Contrasting to the occurring in AD, the role of the adaptive immune system in PD’s pathophysiology is becoming clearer. In fact, numerous authors have found adaptive immune populations, namely Th17 cells, in PD patients’ brain samples (303). In parallel to what is observed for AD, glucose hypometabolism has been implicated in disease pathophysiology, being associated with the development of dementia occurring in the brain cortex (283). Furthermore, PD is characterized by deregulation of several glycolytic enzymes and transporters such as pyruvate dehydrogenase kinase 1 (PDK1), PKM2, LDHA, GLUT1, MCT-1 and MCT-4, as well as increased mitochondrial respiratory activity and oxidative damage in neurons (283).

Recently, PD supplementation with PCs has been drawing attention. Once more, resveratrol exposes its neuroimmunomodulatory properties, being able to suppress microglia activation and decrease the levels of TNF-α, IL-1β and IL-6 and their receptors’ expression in the SNpc of mice with 1-methyl-4-phenyl-1,2,3,6-tetrahydropyridine (MPTP)-induced PD (304). Analogously, curcumin administration has shown to inhibit microglial morphological alterations in an *in vitro* model (305). Curcumin has further demonstrated protective effects against neurodegeneration in the A57Tα-synuclein model of PD by downregulating mTOR/p70S6 kinase (P70S6K) signaling and recovering macro autophagy (306). Accordingly, researchers employing a nanoformulation of α-mangostin discovered that the polyphenol reprogrammes microglia metabolism from glycolysis to OXPHOS and promotes its autophagic capacity, increasing microglial Aβ clearance (307).

Regarding the mitochondrial dysfunction featuring the disease, morin and mangiferin displayed the ability to attenuate membrane potential loss in neurons (308), as well as quercetin which also enhanced mitophagy by upregulating Pink and Parkin gene expression (309).

On another hand, a study evaluating the impact of genistein in dopaminergic neurodegeneration reported a dose-dependent inhibition of neuronal loss in rats’ glial cells (310). The same authors reported the soybean isoflavone’s ability to suppress microglia cell activation as well as NO and superoxide production by these cells (310).

It is worth noting that evidence regarding the importance of the gut-brain and spleen-brain axes in PD has been emerging, suggesting an involvement of the intestinal and splenic immune systems in this disease development (311). Wang et al. evaluated the impact of chicory acid in mice with MPTP-induced PD and verified that this PC prevented dopaminergic brain lesions and glial activation, simultaneously reverting the disease-induced alterations in IL-17, IFN-γ and TGF-β levels in both the spleen and colon (311).

##### 5.2.2.3 Stroke and stroke-induced neurodegeneration

Stroke is one of the global leading causes of disability and mortality (312, 313). It is currently acknowledged that the immune system is an active player in stroke’s pathogenesis, possibly causing subsequent damage which are collectively designated as stroke-induced secondary neurodegeneration (SND), a condition that shares numerous features with AD, namely Aβ accumulation (314).

Similarly to AD and PD, stroke-associated neuroinflammatory events include microglial activation and consequent release of neurotoxic mediators, as well as stimulation of macrophages and DCs (315). Stroke-associated microglial cells display dysfunctional phagocytosis and chemotaxis, severely compromising neuroinflammation resolution and neurorestoration (316). Furthermore, after stroke events activated M1 microglia cells display enhanced mitochondrial fission, leading to NF-kB and MAPK activation which induces pro-inflammatory mediators’ expression (317). These cells have also shown to release damaged mitochondria to neurons where they fuse with neuronal mitochondria, damaging them and promoting mitochondria-mediated neuronal death (318). Additionally, stroke-associated microglia feature increased autophagy in associated with an enhanced inflammatory response (319–321). In agreement, treatment of permanent middle cerebral artery occlusion (pMCAO) mice with an autophagy inhibitor alleviated the inflammatory response, while an autophagy inducer exerted the opposite effect (320).

NK cells and CD4^+^, CD8^+^ and γδ T lymphocytes have likewise revealed to be involved in stroke’s initial stages, with B cell-mediated neurodegeneration becoming prominent later on in the disease course (315). Therefore, components of immunoreactivity can be found in each phase of stroke pathology. Another similarity between AD, PD and stroke is the metabolic reprogramming of microglia cells shifting from OXPHOS to glycolysis (322). In fact, stroke brains display increased concentrations of numerous glycolytic intermediates, including glucose-6-phosphate, fructose-6-phosphate, LDHA, PKM2, pyruvate, and lactate (322). Furthermore, blocking microglial hexokinase-2, the enzyme responsible for glucose phosphorylation into glucose-6-phosphate, has shown to suppress their activation and reduce the infarct area in male Sprague-Dawley rats subjected to transient middle cerebral artery occlusion (323), highlighting the role of glucose metabolism in microglia-mediated neuroinflammation characterizing stroke. Interestingly, the ability of already existing drugs to modulate macrophage and microglia metabolism in the context of stroke is being studied (324). Minocycline, which has exhibited neuroprotective activity in the context of stroke (325), has shown to promote microglia polarization from an M1 to an M2 phenotype through STAT1 and STAT6 pathways (326). Moreover, studies suggest that tissue plasminogen activator (tPA), a widely employed fibrinolytic agent in stroke therapy, is able to normalize microglial chemotaxis and phagocytosis through metabolic pathways’ modulation, including Akt and ERK 1/2 signaling (327, 328).

Likewise, PCs immunomodulatory effects have been demonstrated in situations of stroke and SND as well. For instance, a study evaluating fisetin effects in a mouse model of ischemic stroke highlighted the flavanol’s ability to inhibit post-ischemic infiltration of macrophages and DCs as well as repress the intracerebral activation of immune cells (329). In addition, fisetin shown to suppress TNF-α production by macrophages and microglia cells *in vitro* (329). Regarding PCs’ impact on microglia, a study performed by Lan et al. showed that the flavanone pinocembrin was able to suppress microglia activation and consequent production of IL-6, IL-1β and TNF-α (330), while also decreasing the expression of TLR4 and its downstream target proteins TRIF and myeloid differentiation primary response 88 (MyD88) (330). Similarly, both curcumin (331) and baicalein (332) appeared to ameliorate ischemic brain damage a by modulating microglia polarization and suppressing TLR4 and NF-kB signaling. Gallic acid has also shown to induce microglia M2 polarization in a MCAO mouse model (333).

PCs further seem to impact stroke-associated microglia mitochondria dysfunction, as evidenced by the reduced post-ischemia neuronal mitochondrial damage resulting from kaempferol administration to rat PC12 cells (334). This effect was considered to derive from an upregulated SIRT1 expression alongside to inhibited gene acetylation of the pro-apoptotic protein P66shc as well as Drp1 recruitment.

Remarkably, recent research has demonstrated that consumption of resveratrol after stroke events might exert neuroprotection through gut-brain-axis modulation (335). In fact, the authors determined that the polyphenolic supplementation promoted a polarization shift of Th cells from Th1 to Th2, reducing intestinal inflammation and vascular permeability, which culminated in mitigation of inflammatory brain lesions (335).

## 6 Conclusion and future directions

In recent years, the term “immunometabolism” has gained traction within the scientific and research communities as a descriptor of the interface between the immune system and metabolism. The disruption of such complex interactions is increasingly recognized as a common denominator of a wide range of socioeconomically impactful diseases of both autoimmune and non-autoimmune nature (172). The escalating prevalence of immunometabolic disorders and the intricate interplay between metabolic irregularities and scenarios of chronic inflammation underscore the imperative to unravel the mechanisms that dictate the programming of immune cell metabolism. In fact, cells from the immune system display unique energy requirements depending on their activation state, anabolic and catabolic mechanisms, being associated with pro- and anti-inflammatory responses, respectively (336). Therefore, modulating immune cells’ metabolic pathways through their respective energetic substrates may significantly impact disease outcome. From this perspective, nutritional interventions emerge as promising tools within the realm of preventive and/or adjunct therapeutic approaches, framing the concept of immunonutrition, a branch of precision nutrition aimed to fine tune pro- or anti-inflammatory immunophenotypes through personalized protocols tailored to individual requirements, health status and metabolic variability (57, 337).

Within the context of immunometabolic disorders, polyphenols stand out due to a wealth of evidence supporting their potential health benefits. Remarkably, varied polyphenols have shown to exert immunomodulatory effects, such as curtailing immune cell hyperactivity and rebalancing pro- and anti-inflammatory T cell subsets, naming a few (70). Particularly, these bioactive compounds are reported to influence immune cell metabolic reprogramming, driving a tolerogenic phenotype and mitigating inflammation, thus showcasing significant potential as key immunonutrients. Such outcomes stem from their influence on nutrient-sensing pathways primarily involved in processes like glycolysis, mitochondrial biogenesis and dynamics as well as mitochondria-ER-lysosome inter-organelle connections, leading to epigenetic and metabolic reprogramming that yield diverse immunomodulatory effects across different cell populations as depicted in **Figure 3** (338). It is worth emphasizing that a substantial portion of these regulatory pathways is similarly influenced by the pleiotropic effects of drugs included in current therapeutic algorithms for the aforementioned diseases (as illustrated in **Figure 4**), encouraging further exploration on how to best leverage polyphenols as immunonutrients, including optimal dosing, administration routes and potential drug-nutrients interactions requiring clarification. Likewise, precision nutrition practices must account for the metabolic and immunological changes occurring in various life stages, particularly focusing on aging and associated immunosenescence, an imperative yet unmet need.

Additional gaps surface when one considers clinical trial’s experimental design and the selection of immune biomarkers in studying the efficacy of immunonutrition approaches in the scope of chronic diseases. The intricate nature of nutritional interventions, their multi-target profile, as well as defining control groups, blinding, randomization, and insufficient adherence pose substantial hurdles to study design, results interpretation, and implementation. Overcoming these limitations will undoubtedly improve the level of precision in the clinical application of polyphenols-based immunonutrition and attenuate the massive burden of immunometabolic disorders currently compose. Multi-omics models and the integration of multi-dimensional datasets comprising nutritional genomics, phenotypes and lifestyles are paramount to understand the metabolic variability between individuals and achieve personalized guidance for tailored polyphenol-based immunonutritional plans.

## Author contributions

CF: Writing – review & editing, Writing – original draft, Software, Investigation, Conceptualization. PV: Writing – review & editing. HS: Writing – review & editing. JM: Writing – review & editing, Funding acquisition. MC: Writing – review & editing, Supervision. FR: Writing – review & editing, Validation, Supervision, Funding acquisition, Formal analysis. SV: Writing – review & editing, Writing – original draft, Validation, Supervision, Resources, Project administration, Investigation, Funding acquisition, Formal analysis, Conceptualization.
